# Adult-Onset Foveomacular Vitelliform Dystrophy With Unilateral Presentation: A Case Series

**DOI:** 10.7759/cureus.68214

**Published:** 2024-08-30

**Authors:** Evangelos Spanos, Andreas Roussos, Spyros Atzamoglou, Nikolaos Dimitriou, Ioannis Markopoulos, Efstratios Paroikakis, Dimitris Karagiannis, Vasileios Peponis, Michael Karampelas

**Affiliations:** 1 A’ Ophthalmology Department, Specialized Eye Hospital, Ophthalmiatreio Athinon, Athens, GRC; 2 B’ Ophthalmology Department, Specialized Eye Hospital, Ophthalmiatreio Athinon, Athens, GRC

**Keywords:** best disease, vitelliform, pachychoroid, unilateral, adult-onset, foveomacular, dystrophy

## Abstract

Adult-onset foveomacular vitelliform dystrophy (AOFVD) is a rare condition affecting the macula that presents diagnostic and management challenges due to its varied manifestations and clinical overlap with other retinal disorders. As vitelliform lesions can occur in various conditions, such as Best disease and age-related macular degeneration, clinical presentation, multimodal imaging findings, and genetic testing can aid in accurate diagnosis. Although AOFVD typically affects both eyes, unilateral involvement can occur.

This study presents four cases of unilateral AOFVD in female patients aged 43 to 66 years. Each patient was monitored for two years with fundoscopy and multimodal imaging, including color fundus photography, optical coherence tomography (OCT), OCT-angiography, fluorescein angiography, and fundus autofluorescence (FAF).

All patients presented with a characterized solitary, subfoveal, yellow lesion on fundoscopy. FAF revealed intense hyperautofluorescence corresponding with the lesions. OCT revealed the accumulation of homogenous hyperreflective material between the retinal pigment epithelium and photoreceptors. No abnormal findings were observed in the fellow eyes. Subfoveal choroidal thickness was measured at 355 μm, 545 μm, 486 μm, and 669 μm in the affected eyes.

While AOFVD typically manifests bilaterally, these cases demonstrate a unique unilateral presentation, highlighting the importance of comprehensive examination and differential diagnosis. Distinguishing cases with unilateral presentation from other conditions can be more challenging, so awareness of this unusual phenotype and its clinical characteristics must be raised. Choroidal thickness measurements provide additional insights into AOFVD pathophysiology, suggesting a potential association with the pachychoroid spectrum.

## Introduction

As first described by J Donald Gass in 1974 [1], adult-onset foveomacular vitelliform dystrophy (AOFVD) is a rare macular dystrophy that usually causes significant visual deterioration. The terminology used to describe this condition is diverse, including adult vitelliform macular degeneration, adult-onset foveomacular pigment epithelial dystrophy, pseudovitelliform macular degeneration, adult vitelliform macular dystrophy, and adult foveomacular vitelliform dystrophy. AOFVD belongs to the heterogeneous group of pattern dystrophies (PDs) that also includes other conditions such as reticular dystrophy of the retinal pigment epithelium (RPE), pseudo-Stargardt pattern dystrophy, fundus pulverulentus, and butterfly-shaped pigment dystrophy [2].

AOFVD usually manifests between 30 and 50 years of age, though it can vary. In fundoscopy, the condition initially appears as a round or oval, symmetrical, yellowish, slightly elevated lesion covering one-third to one-half disc diameter in size. On optical coherence tomography (OCT), it appears as a dome-shaped hyperreflective sub-neuroretinal lesion, which initially has uniform hyperreflectivity but several hyporeflective areas appear during evolution. On fundus autofluorescence (FAF), the yellow lesion exhibits intense hyperautofluorescence. The deposits that compose the lesion are of extracellular photoreceptor outer segment debris, pigment and lipofuscin-laden RPE cells, and macrophages. Over the years, the lesion can grow in size, lose its original shape, and eventually start to break up, leading to geographical atrophy, similar to age-related macular degeneration (AMD) [2]. At late stages, choroidal neovascularization and fibrosis can occur, which can result in severe visual loss.

A variety of imaging tools have been used to study the disease. Gass originally used fluorescein angiography (FA) to describe his findings, while indocyanine green angiography (ICGA) and FAF have also been used to determine the shape and progression of the lesions [1,2]. To assess the functionality of the RPE, electrophysiological studies, including electro-oculography (EOG), have been used [3]. Additionally, recent reports relate AOFVD with increased choroidal thickness [4-6].

Several genes have been related to the disease, including mutations in *PRPH2*, *BEST1*, *IMPG1*, or *IMPG2* [7,8]. Because these genes seem to appear in other related macular degenerations such as Best disease, there is no clear unique correlation between the aforementioned genes and AOFVD.

AOFVD most commonly presents bilaterally, though there are case reports with unilateral involvement [9,10]. We present four cases of unilateral AOFVD. Two of the cases were newly diagnosed, while the other two cases refer to patients with a known history of maculopathy. All cases were monitored with fundoscopy and multimodal imaging for two years.

## Case presentation

Case 1

**Figure 1:**
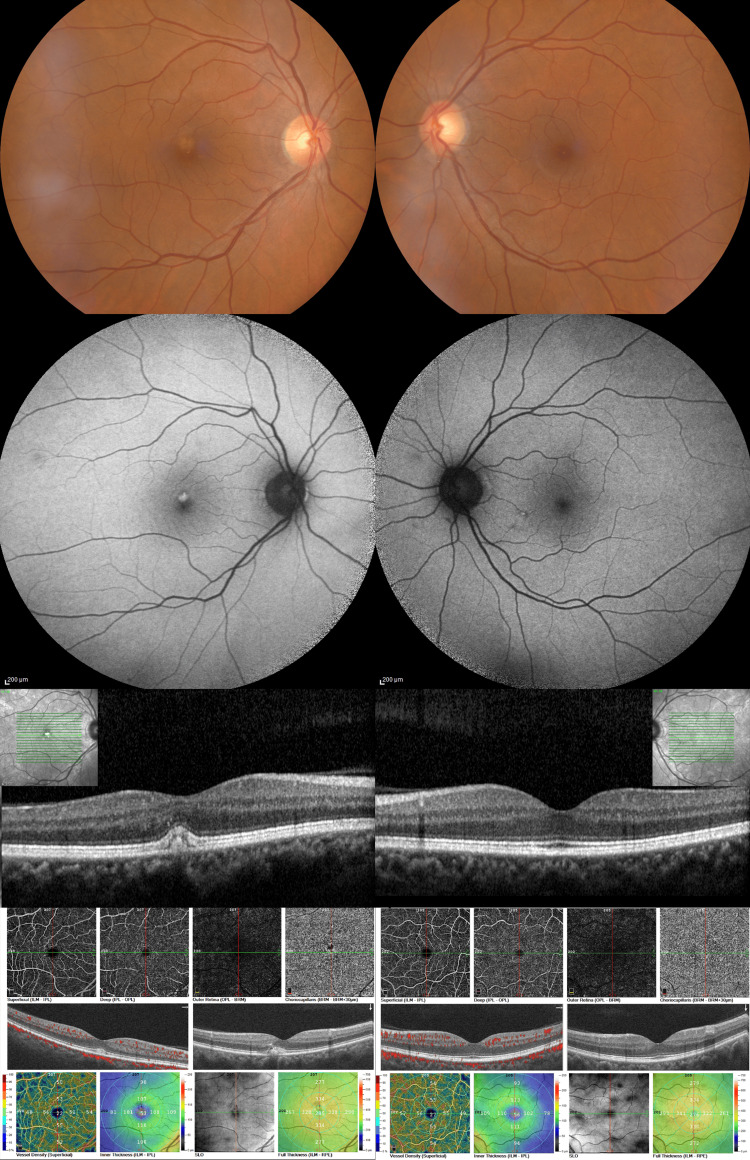
Multimodal imaging of the right eye (left column) and the left eye (right column) of patient 1. Color fundus photographs (top row), fundus autofluorescence images (second row), horizontal spectral-domain optical coherence tomography (OCT) B-scans (third row), and OCT-angiography (OCT-A) images (bottom row). The right eye demonstrates a typical vitelliform lesion at the central macula that exhibits bright hyperautofluorescence and corresponds to sub-neuroretinal homogenous hyperreflective material on OCT. OCT-A reveals a central area of blockage corresponding with the vitelliform lesion.

A 51-year-old female patient presented at the emergency department complaining of a unilateral decrease in her visual acuity. No past ocular or systemic comorbidities were reported and the family medical history was unremarkable. At presentation, the best-corrected visual acuity (BCVA) was 8/10 and 10/10 (OD and OS, respectively). The anterior-segment examination was unremarkable. Fundoscopy revealed a single, yellow/orange, well-circumscribed, dome-shaped, egg-yolk-like subfoveal lesion in the right eye (Figure 1). OCT revealed an accumulation of homogenous hyperreflective material between the RPE and photoreceptors (Figure 1). FAF revealed intense hyperautofluorescence corresponding with the lesion (Figure 1). Enhanced depth imaging OCT (EDI-OCT) was performed at the subfoveal region. Subfoveal choroidal thickness (SCT) was 355 μm on the affected eye and 318 μm on the fellow eye. OCT-angiography (OCT-A) did not reveal any other abnormality but did demonstrate a dark area corresponding with the lesion in all retinal slabs due to blockage from the vitelliform material (Figure 1). Fundoscopy and multimodal imaging of the left eye were unremarkable (Figure 1).

Case 2

**Figure 2:**
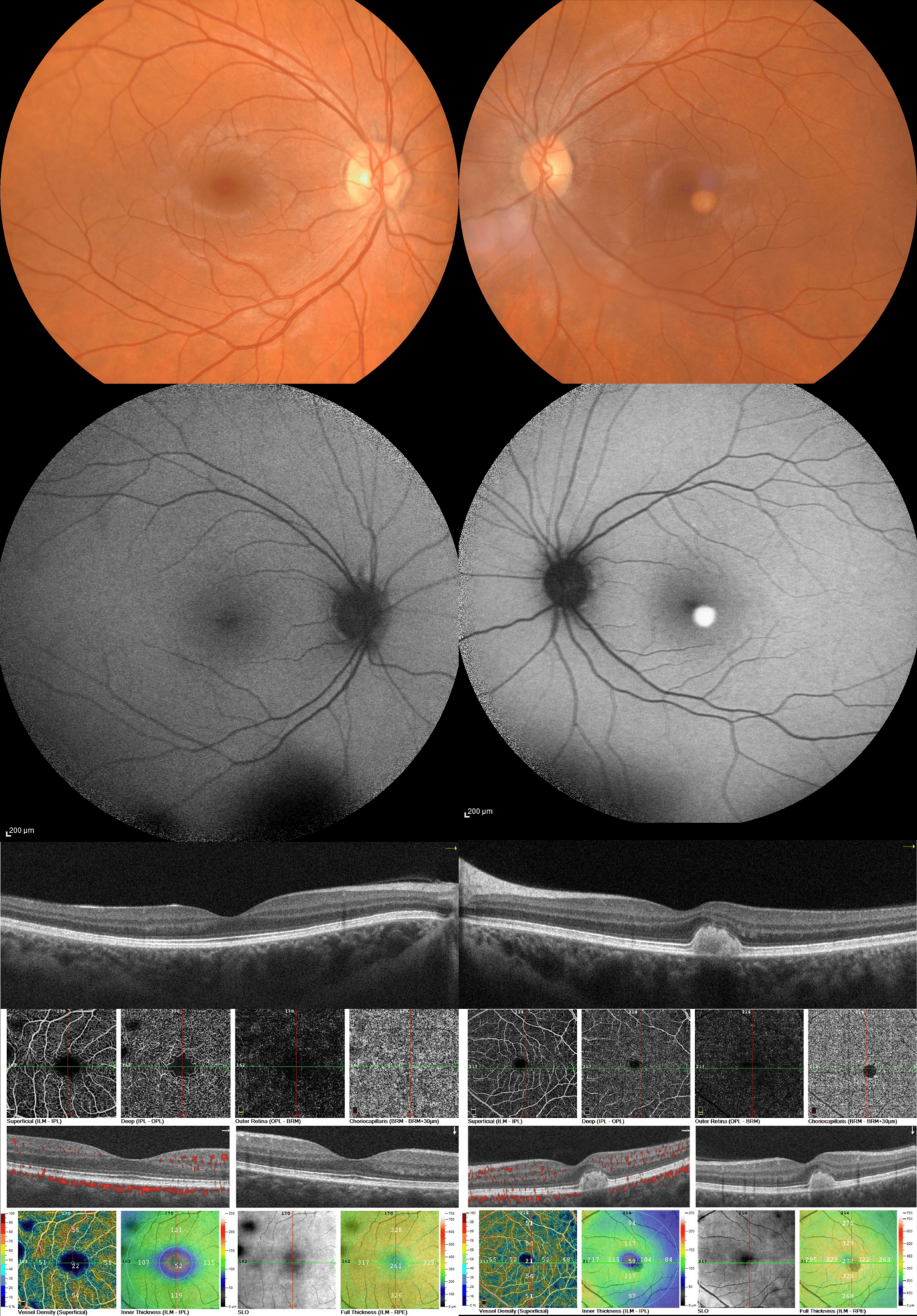
Multimodal imaging of the right eye (left column) and the left eye (right column) of patient 2. Color fundus photographs (top row), fundus autofluorescence images (second row), horizontal spectral-domain optical coherence tomography (OCT) B-scans (third row), and OCT-angiography (optical coherence tomography (OCT)-A) images (bottom row). The left eye demonstrates a typical vitelliform lesion at the central macula that exhibits bright hyperautofluorescence and corresponds to sub-neuroretinal homogenous hyperreflective material on OCT. OCT-A reveals a central area of blockage corresponding with the vitelliform lesion. Choroids appear thickened bilaterally.

A 43-year-old female patient presented at the emergency department complaining of blurred vision and metamorphosia for the past few months. No past medical history was reported. Her BCVA was 10/10 and 9/10 (OD and OS, respectively). Fundoscopy examination revealed a yellow/orange, well-circumscribed, dome-shaped, egg-yolk-like lesion under the fovea of the left eye which presented hyperreflective on OCT and bright hyperautofluorescent on FAF (Figure 2). SCT was measured at 666 μm and 545 μm (OD and OS, respectively). OCT-A demonstrated a dark area corresponding with the lesion in all retinal slabs due to blockage from the vitelliform material (Figure 2). Fundoscopy and multimodal imaging of the right eye did not reveal any significant findings (Figure 2).

Case 3

**Figure 3:**
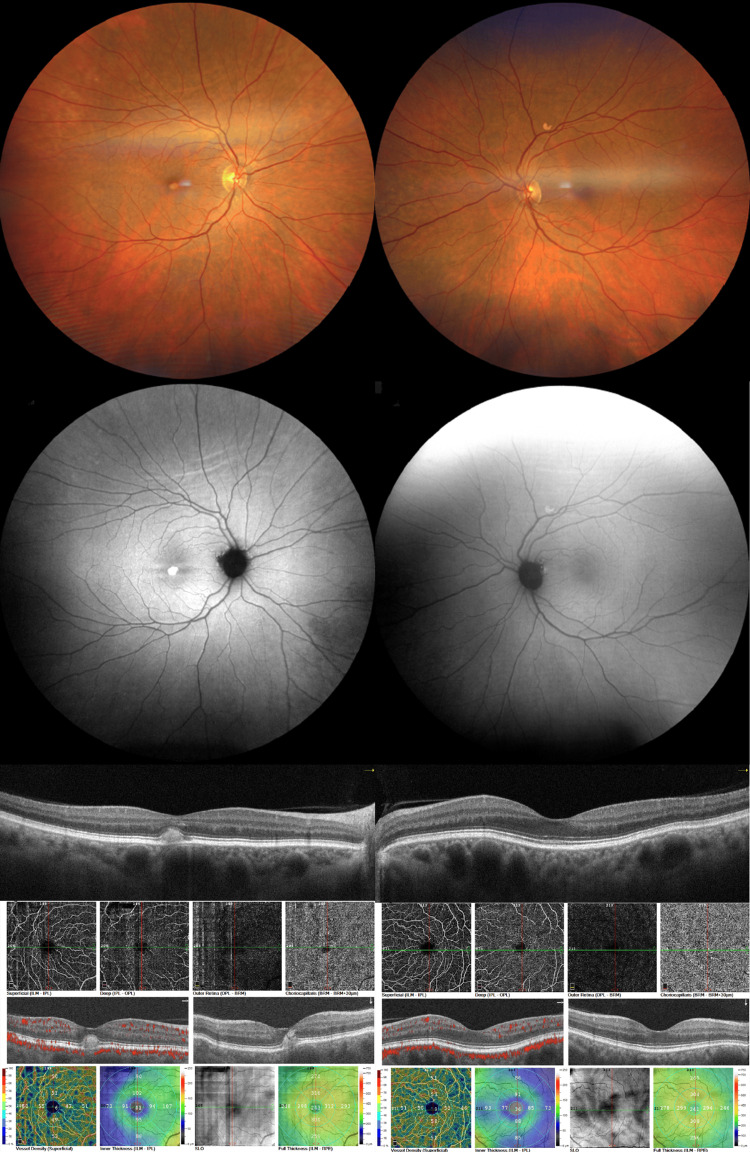
Multimodal imaging of the right eye (left column) and the left eye (right column) of patient 3. Color fundus photographs (top row), fundus autofluorescence images (second row), horizontal spectral-domain optical coherence tomography (OCT) B-scans (third row), and OCT-angiography (OCT-A) images (bottom row). The right eye demonstrates a typical vitelliform lesion at the central macula that exhibits bright hyperautofluorescence and corresponds to sub-neuroretinal homogenous hyperreflective material on OCT. OCT-A reveals a central area of blockage corresponding with the vitelliform lesion. The left eye demonstrates a pachy-drusen above the superior temporal arcade that exhibits hyper-autofluorescence. Choroids appear thickened bilaterally.

A 66-year-old female patient presented to our retina department with a history of maculopathy in the right eye. Past medical history included deteriorating vision in the affected eye during the last five years. BCVA was 8/10 and 9/10 (OD and OS, respectively). The anterior-segment examination was unremarkable bilaterally. Fundoscopy revealed a subfoveal, round, yellow lesion on the right eye (Figure 3). OCT demonstrated a subfoveal hyperreflective material accumulation with a mild disruption of the outer retinal layers in a vitelliruptive-like configuration (Figure 3). FAF revealed intense hyperautofluorescence corresponding with the lesion (Figure 3). SCT was measured at 486 μm and 439 μm (OD and OS, respectively). OCT-A of the right eye revealed a central area of blockage corresponding with the vitelliform lesion (Figure 3). Fundoscopy and multimodal imaging of the left eye demonstrated a single pachy-drusen above the superior temporal arcade that exhibited hyperautofluorescence (Figure 3).

Case 4

**Figure 4:**
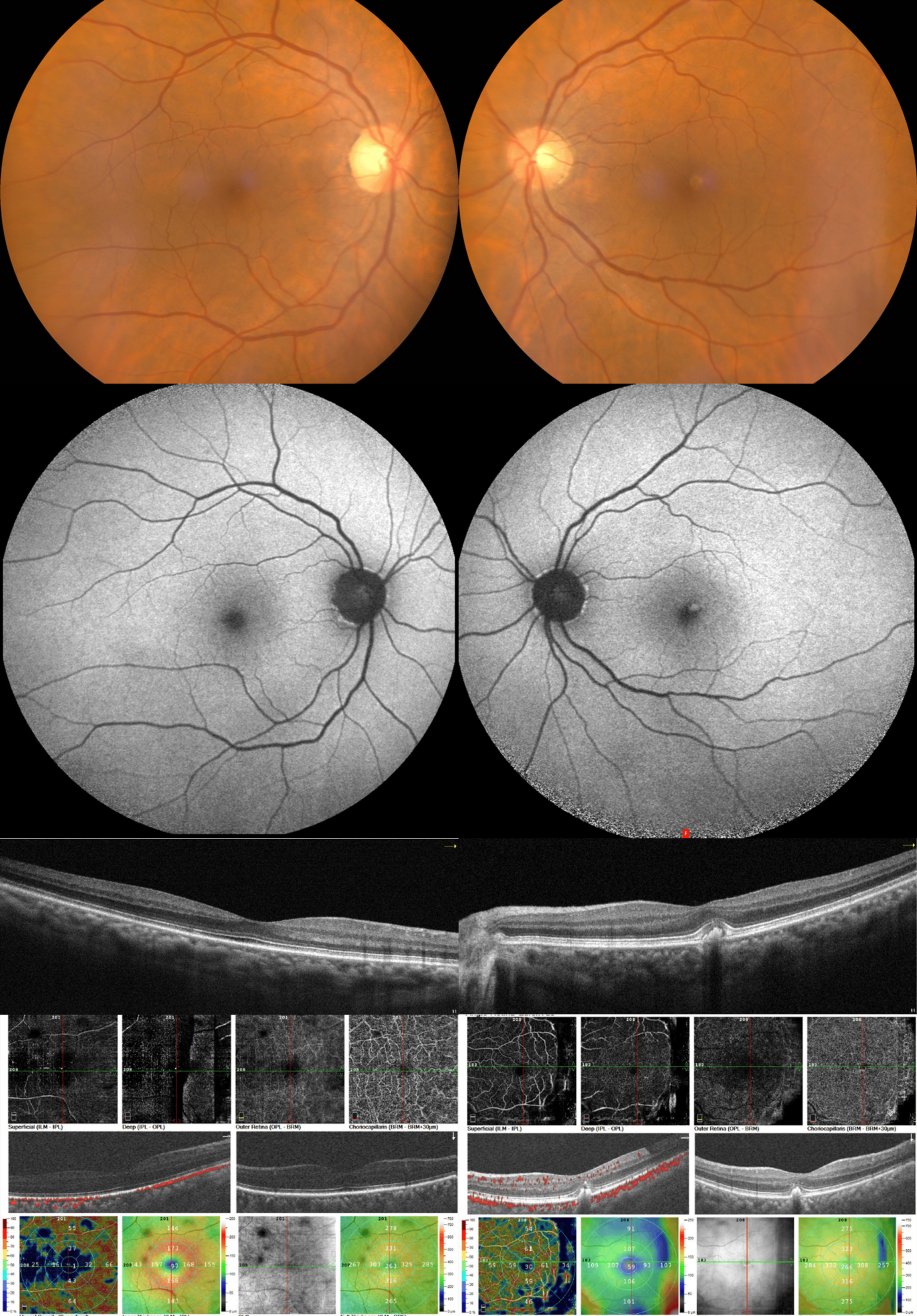
Multimodal imaging of the right eye (left column) and the left eye (right column) of patient 4. Color fundus photographs (top row), fundus autofluorescence images (second row), horizontal spectral-domain optical coherence tomography (OCT) B-scans (third row), and OCT-angiography (OCT-A) images (bottom row). The left eye demonstrates a vitelliform lesion at the central macula that exhibits hyperautofluorescence and corresponds to sub-neuroretinal homogenous hyperreflective material on OCT. OCT-A reveals a central area of blockage corresponding with the vitelliform lesion.

A 64-year-old female patient was referred to the retina department for further evaluation due to a macula disorder. BCVA was 10/10 and 9/10 (OD and OS, respectively). A single, round, yellow subfoveal lesion was found during fundoscopy on the left eye (Figure 4). The lesion presented hyperreflective on OCT and mildly hyperautofluorescent on FAF (Figure 4). On EDI-OCT the SCT was measured to be 336 μm and 668 μm (OD and OS, respectively). OCT-A of the left eye revealed a central area of blockage corresponding with the vitelliform lesion. Fundoscopy and multimodal imaging of the right eye was unremarkable (Figure 4).

During the two-year follow-up, no significant changes were observed in the affected or the contralateral eyes in all patients.

## Discussion

AOFVD-like lesions can be associated with several pathologies; thus, a careful examination is essential and a detailed medical history must be obtained. Best disease is an autosomal dominant retinal disorder with variable expression and penetrance. Best disease also exhibits varying heterogeneity, producing different phenotypes with clinical features that overlap with AOFVD, making these two entities often indistinctive. Best disease typically has a younger age of onset and patients often present with larger and multifocal vitelliform lesions. EOG is usually subnormal in Best disease, unlike AOFVD in which EOG is typically not affected [2]. *BEST1* gene mutations are always present in Best disease but only in a minority of patients with AOFVD. Several other genes (*IMPG1*, *IMPG2*, *PRH1*) have been associated with AOFVD; however, in most cases, no genetic cause is identified [2]. Additionally, while Best disease predominately affects both eyes, a unilateral presentation has also been described [7,8,11,12]. Considering our patients’ age of onset, the solitary small-sized lesion, the unilateral manifestation, the lack of associated family history, and the absence of similar lesions in the fellow eyes during follow-up, it was concluded that these cases represent a unilateral presentation of AOFVD than an atypical presentation of late-onset Best’s macular dystrophy.

Distinguishing AOFVD from AMD can be quite challenging. Vitelliform lesions may appear in a variety of drusen types, including the ones that are typical of AMD [2]. On the other hand, due to the similar age of onset, drusenoid deposits may be found in patients who developed vitelliform lesions, in the context of AOFVD. In the reported cases, no typical signs of AMD were found in fundoscopy or multimodal imaging. Similar to dry AMD, visual acuity is usually well preserved during the course of the disease and the patients are only mildly symptomatic, until the latter stages. Choroidal neovascularization (CNV) development has been reported to occur in 5-15% of AOFVD patients [13], and in these cases, OCT-A is a helpful tool in CNV detection [14-17]. During follow-up, none of the reported patients developed CNV.

Except for Best disease and AMD, several other entities may lead to vitelliform lesions and have to be differentiated from AOFVD. Conditions that cause a chronic separation of the RPE and photoreceptors may also result in vitelliform material accumulation. These include chronic serous chorioretinopathy, epiretinal membrane, and vitreomacular traction. Numerous systemic disorders, drug toxicity, trauma, and other inherited disorders, such as autosomal recessive bestrophinopathy and butterfly PD, can also lead to vitelliform lesions [2]. Unlike Best disease and AMD, distinguishing AOFVD from these entities can be easier. Our reported cases had an unremarkable ocular, general, and family history and a strikingly unilateral presentation. Furthermore, no additional clinical findings were noticed, related to any other condition.

To our knowledge, very few cases of unilateral AOFVD have been described in the literature. Georgiou et al. reported a similar case of unilateral AOFVD, associated with an *IMPG2* gene variant [9]. A solitary subfoveal yellow lesion was described, hyperautofluorescent on FAF, and with increased reflectivity on OCT. A 10-year follow-up of this patient revealed only progressive drusenoid changes, without bilateral involvement. Subash et al. described six unrelated cases in which a similar unilateral AOFVD phenotype was identified [10]. Five cases had an isolated, small, yellow subretinal lesion with corresponding findings on OCT and FAF. One patient presented with a vitelliruptive-like configuration and significant visual impairment. All patients were screened for *BEST1* and *PRPH2* pathogenic mutations, in which no molecular correlation was identified for this unilateral phenotype.

Until now, electrophysiological studies have not shown any definite findings in AOFVD. Only a few cases have shown a slightly abnormal EOG which indicates that the condition does not seem to be related to a widespread generalized RPE dysfunction [3].

Choroidal thickness varies with age, refractive status, and certain pathologies [18,19]. In AMD, submacular choroidal thickness is decreased compared to normal subjects [20]. Entities under the pachychoroid spectrum present with increased choroidal thickness [21]. Several studies have reported subfoveal choroidal thickness in normal eyes. In two large series, Pongsachareonnont et al. reported a mean subfoveal choroidal thickness of 265.5 ± 74.2 μm, while Ruiz-Medrano et al. reported a mean thickness of 301.89 μm [22,23]. There are several conflicting reports regarding choroidal thickness in patients with AOFVD. Grenga et al. reported increased subfoveal choroidal thickness in the vitelliruptive stage compared to the control group, though no significant differences were reported through the other stages of the disease [4]. Coscas et al. reported increased thickness in patients with AOFVD in eyes regardless of fluid presence compared to the control group [5]. In another study by Palacios et al., subfoveal and mean choroidal thickness presented no significant difference [6]. AOFVD has been reported in patients with pachychoroid, either asymptomatic or with clinical manifestations of the disorders falling under the pachychoroid spectrum [24-26]. Researchers have thus suggested the term pachyvitelliform [27]. It is not yet clear whether AOFVD is associated with this condition or is an accidental finding. SCT was found to be significantly increased compared to the reported normal range in two of our patients in the affected as well as their contralateral eyes (545-666 μm and 486-439 μm in Case 2 and Case 3, respectively). Additionally, in one of our cases, SCT was found to be significantly increased in the affected eye (668 μm) and within the normal range (336 μm) in the contralateral eye. There were no signs of active disease under the pachychoroid spectrum in either eye but a single extramacular pachydrusen was found in the healthy eye of Case 3. To our knowledge, these are the first cases reported in the literature with unilateral AOFVD and pachychoroid, without clinical manifestation from the latter.

One of the limitations of our study is that the cases were monitored for a two-year period; therefore, a late onset involvement of the fellow eye cannot be excluded. In addition, gene testing was not performed due to the unwillingness of the patients. Although there is no clear unique correlation between specific gene mutations and AOFVD, gene testing could indicate other causes of vitelliform material accumulation, such as Best disease.

## Conclusions

Although AOFVD predominately presents with typical clinical findings, it demonstrates great clinical variability. Additionally, vitelliform lesions can also occur in several other conditions producing overlapping phenotypes with AOFVD. This complexity underscores the importance of thorough examination and consideration of differential diagnoses, including Best disease and AMD. Specific features such as age of onset, lesion size, family history, and associated findings aid in diagnosis. Multimodal imaging plays a crucial role in diagnosis and monitoring, offering insights into disease progression and complications such as CNV. Distinguishing cases with unilateral presentation from other conditions can be more challenging, so awareness of this unusual phenotype and its clinical characteristics must be raised. Three of our cases demonstrated increased subfoveal choroidal thickness, a finding that warrants further investigation into the relationship between AOFVD and pachychoroid spectrum.

## References

[1] Gass JD (1974). A clinicopathologic study of a peculiar foveomacular dystrophy. Trans Am Ophthalmol Soc.

[2] Chowers I, Tiosano L, Audo I, Grunin M, Boon CJ (2015). Adult-onset foveomacular vitelliform dystrophy: a fresh perspective. Prog Retin Eye Res.

[3] Renner AB, Tillack H, Kraus H (2004). Morphology and functional characteristics in adult vitelliform macular dystrophy. Retina.

[4] Grenga PL, Fragiotta S, Cutini A, Meduri A, Vingolo EM (2016). Enhanced depth imaging optical coherence tomography in adult-onset foveomacular vitelliform dystrophy. Eur J Ophthalmol.

[5] Coscas F, Puche N, Coscas G (2014). Comparison of macular choroidal thickness in adult onset foveomacular vitelliform dystrophy and age-related macular degeneration. Invest Ophthalmol Vis Sci.

[6] Palácios RM, Mendes TS, Sano RY, Wu DC, Aihara T, de Almeida Manzano RP (2016). Choroidal thickness using EDI-OCT in adult-onset vitelliform macular dystrophy. Int J Retina Vitreous.

[7] Kaden TR, Tan AC, Feiner L, Freund KB (2017). Unilateral Best disease: a case report. Retin Cases Brief Rep.

[8] Wabbels B, Preising MN, Kretschmann U, Demmler A, Lorenz B (2006). Genotype-phenotype correlation and longitudinal course in ten families with Best vitelliform macular dystrophy. Graefes Arch Clin Exp Ophthalmol.

[9] Georgiou M, Chauhan MZ, Michaelides M, Uwaydat SH (2022). IMPG2-associated unilateral adult onset vitelliform macular dystrophy. Am J Ophthalmol Case Rep.

[10] Subash M, Rotsos T, Wright GA (2012). Unilateral vitelliform maculopathy: a comprehensive phenotype study with molecular screening of BEST1 and PRPH2. Br J Ophthalmol.

[11] Arora R, Khan K, Kasilian ML (2016). Unilateral BEST1-associated retinopathy. Am J Ophthalmol.

[12] Querques G, Zerbib J, Santacroce R (2009). Functional and clinical data of Best vitelliform macular dystrophy patients with mutations in the BEST1 gene. Mol Vis.

[13] Da Pozzo S, Parodi MB, Toto L, Ravalico G (2001). Occult choroidal neovascularization in adult-onset foveomacular vitelliform dystrophy. Ophthalmologica.

[14] Stattin M, Ahmed D, Glittenberg C, Krebs I, Ansari-Shahrezaei S (2020). Optical coherence tomography angiography for the detection of secondary choroidal neovascularization in vitelliform macular dystrophy. Retin Cases Brief Rep.

[15] Querques G, Zambrowski O, Corvi F, Miere A, Semoun O, Srour M, Souied EH (2016). Optical coherence tomography angiography in adult-onset foveomacular vitelliform dystrophy. Br J Ophthalmol.

[16] Joshi KM, Nesper PL, Fawzi AA, Mirza RG (2018). Optical coherence tomography angiography in adult-onset foveomacular vitelliform dystrophy. Retina.

[17] Lupidi M, Coscas G, Cagini C, Coscas F (2015). Optical coherence tomography angiography of a choroidal neovascularization in adult onset foveomacular vitelliform dystrophy: pearls and pitfalls. Invest Ophthalmol Vis Sci.

[18] Abbey AM, Kuriyan AE, Modi YS (2015). Optical coherence tomography measurements of choroidal thickness in healthy eyes: correlation with age and axial length. Ophthalmic Surg Lasers Imaging Retina.

[19] Yavuzer K, Bozkurt B, Turgut Ozturk B (2021). The effect of age on subfoveal choroidal thickness in healthy subjects. Eastern J Med.

[20] Salehi MA, Mohammadi S, Gouravani M, Rezagholi F, Arevalo JF (2023). Retinal and choroidal changes in AMD: a systematic review and meta-analysis of spectral-domain optical coherence tomography studies. Surv Ophthalmol.

[21] Cheung CM, Lee WK, Koizumi H, Dansingani K, Lai TY, Freund KB (2019). Pachychoroid disease. Eye (Lond).

[22] Pongsachareonnont P, Somkijrungroj T, Assavapongpaiboon B, Chitamara T, Chuntarapas M, Suwajanakorn D (2019). Foveal and parafoveal choroidal thickness pattern measuring by swept source optical coherence tomography. Eye (Lond).

[23] Ruiz-Medrano J, Flores-Moreno I, Peña-García P, Montero JA, Duker JS, Ruiz-Moreno JM (2014). Macular choroidal thickness profile in a healthy population measured by swept-source optical coherence tomography. Invest Ophthalmol Vis Sci.

[24] Soman M, Iqbal S, Sheth JU, Meleth P, Nair U (2021). Progression of subclinical pachychoroid neovasculopathy to an active neovascularization in the presence of acquired vitelliform lesions. Case Rep Ophthalmol Med.

[25] Barequet D, Iglicki M, Meshi A, Loewenstein A, Goldstein M, Zur D (2022). Acquired vitelliform lesions: a novel finding in eyes with peripapillary pachychoroid syndrome. Retina.

[26] Garg E, Essilfie J, Sacconi R, Querques G, Sarraf D (2018). Acquired vitelliform maculopathy in pachychoroid pigment epitheliopathy and comparison to acquired vitelliform maculopathy in reticular drusen. Invest Ophthalmol Vis Sci.

[27] Hilely A, Au A, Lee WK (2024). Pachyvitelliform maculopathy: an optical coherence tomography analysis of a novel entity. Br J Ophthalmol.

